# LncRNA *FIRRE* functions as a tumor promoter by interaction with PTBP1 to stabilize BECN1 mRNA and facilitate autophagy

**DOI:** 10.1038/s41419-022-04509-1

**Published:** 2022-02-02

**Authors:** Yajie Wang, Zhengyang Li, Shizan Xu, Wenjun Li, Mengyun Chen, Miao Jiang, Xiaoming Fan

**Affiliations:** 1 Department of Gastroenterology, Jinshan Hospital, Fudan University, 1508 Longhang Road, Shanghai, 201508 China; 2 Department of Anaesthesiology, Shanghai Public Health Clinical Center, Fudan University, 2901 Caolang Road, Shanghai, 201503 China; 3 General Practice of Huamu Community Health Service Center, 90 Yulan Road, Shanghai, 201204 China

**Keywords:** Cancer genomics, Oncogenes

## Abstract

Long non-coding RNAs (lncRNAs) play critical functions in various cancers. Firre intergenic repeating RNA element (FIRRE), a lncRNA located in the nucleus, was overexpressed in colorectal cancer (CRC). However, the detailed mechanism of FIRRE in CRC remains elusive. Results of RNA sequence and qPCR illustrated overexpression of FIRRE in CRC cell lines and tissues. The aberrant expression of FIRRE was correlated with the migration, invasion, and proliferation in cell lines. In accordance, it was also associated with lymphatic metastasis and distant metastasis in patients with CRC. FIRRE was identified to physically interact with Polypyrimidine tract-binding protein (PTBP1) by RNA pull-down and RNA immunoprecipitation (RIP). Overexpression of FIRRE induced the translocation of PTBP1 from nucleus to cytoplasm, which was displayed by immunofluorescence and western blot. In turn, delocalization of FIRRE from nucleus to cytoplasm is observed after the loss of PTBP1. The RNA-protein complex in the cytoplasm directly bound to BECN1 mRNA, and the binding site was at the 3' end of the mRNA. Cells with FIRRE and PTBP1 depletion alone or in combination were treated by Actinomycin D (ACD). Results of qPCR showed FIRRE stabilized BECN1 mRNA in a PTBP1-medieated manner. In addition, FIRRE contributed to autophagy activity. These findings indicate FIRRE acts as an oncogenic factor in CRC, which induces tumor development through stabilizing BECN1 mRNA and facilitating autophagy in a PTBP1-mediated manner.

## Introduction

Colorectal cancer (CRC) is the third most prevalent cancer in the world, leading to 53,200 deaths per year [1]. In China, the estimated number of new CRC diagnosis is over 380,000 annually [2]. The high death rate and limited therapeutic options warrant further research on CRC regulatory mechanisms of great significance.

The development of CRC is a complicated biological process, accompanied by multiple alterations in protein-coding and non-protein-coding genes [3, 4]. Among non-coding genes, lncRNAs have been proved to play crucial roles in CRC [5, 6]. Interaction between lncRNAs and proteins are widely involved in the pathological processes of tumors through a variety of mechanisms [7], including regulating RNA stability [8], variable splicing [9], and protein stability [10]. FIRRE is a conserved lncRNA that localizes on the X chromosome [11]. Few studies have been performed to investigate effect of FIRRE on cancers. FIRRE was identified to be significantly associated with poor OS of neuroblastoma [12] and function as an oncogene by promoting cell proliferation and reducing cell apoptosis in Diffuse large B-cell lymphoma (DLBCL) [13]. Nevertheless, the exact function and molecular mechanism of FIRRE in CRC are unclear.

Polypyrimidine tract-binding protein (PTBP1, also known as hnRNP I), a member of the hnRNPs family, is found to be elevated in CRC and associated with poor outcomes [14]. As an RNA-binding protein, PTBP1 has been validated to interact with lncRNAs to regulate various biological processes [15]. HnRNP U, a member of the hnRNPs family, was physically bound with FIRRE RNA^7^, indicating the possible interaction between PTBP1 and FIRRE. PTBP1 is capable of shuttling from the nucleus to the cytoplasm [16]. The subcellular localization is essential for the function of PTBP1. Cytoplasmic localization of PTBP1 is required for all steps of mRNA metabolism, including transport and mRNA stabilization [17].

Autophagy is a double-edged sword role in cancer and is involved in the occurrence, maintenance, and development of cancer [18]. Autophagy sometimes acts as a tumor promoter under stress conditions [19, 20]. BECN1 is a key factor in autophagy and its roles in CRC are still unclear. BECN1 served as a negative regulator of CRC metastasis in Hu’s research, suggesting that BECN1 is a tumor suppressor [21]. On the contrary, it has also been reported that BECN1 is closely linked to colorectal carcinogenesis and distant metastasis of colorectal carcinoma [22]. These studies suggest that BECN1 plays different roles in cancer, which requires further exploration.

Here, our research aims to investigate the FIRRE expression in CRC patients. We also explore the roles of FIRRE in CRC and detailed mechanism in FIRRE reprogramming cell lines. Furthermore, we attempt to clarify the relationship between FIRRE and autophagy.

## Methods

### Subjects and specimens

A total of 74 paired CRC tissues and adjacent normal tissues were collected from patients who underwent radical resection at the Jinshan Hospital Fudan University (Shanghai, China) between 2015 and 2017. All the participants were histologically diagnosed as colorectal adenocarcinoma by pathologists. This study was approved by the Ethical Committee of Jinshan Hospital Fudan University, which complies with the code of Ethics of the World Medical Association (Declaration of Helsinki). All collected tissue samples were immediately stored at −80 °C for RNA extraction.

### HE staining and analysis

The pathologic information of all samples was determined by two experienced pathologists, and HE stains were performed according to the standard method [23].

### Cell culture

Human colorectal carcinoma cell lines RKO, HCT116, HT-29, human normal colon epithelial cell line FHC and 293 T were cultured in Dulbecco’s modified Eagle’s medium (DMEM, Cat#11965092, Gibco, USA) with 10% fetal bovine serum (FBS, Cat#16140071, Gibco, USA) and 1% penicillin and streptomycin (Cat#KGY0023, KeyGEN BioTECH, China). SW480 and SW620 were maintained in Leibovitz’s L-15(Cat#11415114, Gibco, USA) supplemented with 10% FBS and 1% penicillin and streptomycin (Cat#KGY0023, KeyGEN BioTECH, China). All cells were maintained at 37 °C with 5% CO2. The cell lines were donated by the State Key Laboratory of Oncogenes and Related Genes of Shanghai Cancer Institute, and were recently authenticated by STR profiling and tested for mycoplasma contamination.

### RNA extraction and qRT-PCR analyses

Total RNA was extracted using TRIzol (Cat#15596018, Invitrogen, USA) and cDNA was generated according to a reverse transcription system from Takara. Quantitative real-time PCR (qRT-PCR) was performed using the SYBR Green PCR kit (Cat#RR420L, Takara, Japan) with GAPDH as an internal control for normalization. Primer sequences used for qRT-PCR are listed in Supplementary Table 1.

### Plasmid construction, lentivirus production, and infection

The positive recombinant pIRES2-EGFP-FIRRE vector, pMD2.G, and pSPSX2 plasmid (presented by the Department of urinary surgery of Jinshan Hospital) were co-transfected into HEK-293T cells to construct a lentivirus vector. The supernatant was collected after the cells were cultured for 24, 48, and 72 h, respectively. For infection, RKO and HCT116 cells were incubated with lentiviruses for 12 h before washing the excess virus. Forty-eight hours after infection, Puromycin (0.5 μg/mL) (Cat#540411, Sigma, USA) was added to the medium for selection.

Plasmids were also constructed for in vitro RNA transcription. The pUC19 (Purchased from Beyotime Biotechnology, Shanghai, China) acted as empty vector combining with the sense and antisense FIRRE. Subsequently, the Plasmids Transformation was performed using DH5α competent cells (Cat#9057, Takara, Japan) and cultured in LB Nutrient Agar Medium supplemented 0.1% ampicillin (Cat#A5354, Sigma, USA) overnight. After amplification, extraction (Cat#DP118, TIANGEN, China), and purification (Cat#DP204, TIANGEN, China), the plasmids were prepared for in vitro RNA transcription.

### Transient siRNA and antisense oligonucleotides (ASO) transfection

For transient knockdown experiments, the target sequences of siRNAs for PTBP1 were purchased from GenePharma (Shanghai, China) and the target sequences of ASO for FIRRE were purchased from RiboBIO (Guangzhou, China). Cells with confluence >60% were co-incubated with non-silencing control or targeting siRNAs/ASO. Cells were plated 24 h prior to transfections. On the day of transfection, media was switched to Opti-MEM (Cat#11058021, Gibco, USA) and transfection complexes were prepared with 50 nM of siRNAs/ASO following manufactures procedures. The transfection reagent is Lipo3000 (Cat#L3000015, Invitrogen, USA). The effect of the knockdown was measured by qRT-PCR and western blot after 48-72 h of transfection. Sequences of siRNA for PTBP1 knockdown, ASO for FIRRE knockdown are listed in Supplementary Table 2.

### Invasion/migration assay

The 24-transwell migration chambers (8 μm pore) (Cat#3464, Corning Life Sciences, USA) were used to assess migration and invasion ability, coated with BD Matrigel^TM^ Basement Membrane Matrix (Cat#354234, BD Biosciences, USA) (1:1 dilution, 50 μl/cm2) or not. The serum-free medium was in the upper wells while the medium with 20% FBS was in the bottom wells. Ten thousand cells were incubated in the upper wells for 48 h, then transwell chambers were fixed by 4% paraformaldehyde (Cat#KGIHC007, KeyGEN BioTECH, China) and stained with crystal violet (Cat#94448, Sigma, USA).

### Cell proliferation assay

The RKO and HCT116 cells with FIRRE overexpression or depletion were plated into 96-well plates (1*10^4^ cells/well) and incubated overnight. The proliferation ability of cells was assessed by CCK-8 (Cat#ck04, Dojindo Laboratories, Japan) assay. The absorbance at 450 nm was measured every 12 h.

### Colony growth assay

About 1000 CRC cells were incubated in plates with six wells for a week. After being fixed by 4% paraformaldehyde (Cat#KGIHC007, KeyGEN BioTECH, China) and stained by crystal violet (Cat#94448, Sigma, USA), colony number was counted.

### Cell cycle assay

A cell cycle assay was performed in cells with FIRRE silence; Cells were collected and fixed by 70% ethanol at 4 °C overnight. According to the manufacturer’s instructions, propidium iodide (PI) (Cat#550825, BD Biosciences, USA) was applied to stain DNA and flow cytometer was used to analyze cell cycle.

### In vitro RNA transcription and purification of RNA or DNA

RNA probes were transcribed in vitro from linear pUC19 cut by the Xba/restriction enzyme. RNAs were synthesized using the RiboMAX™ Large Scale RNA Production Systems–SP6 and T7 (Cat#P1300, Promega, USA) according to the manufacturer’s protocol. RNA sequences were treated with RNase-free DNase I and purified with the RNA purification kit (Cat#DP412, TIANGEN, China). The quantity of the RNAs was assessed by NanoDrop spectrophotometer and the quality was checked visually on a 1% TAE gel.

### RNA immunoprecipitation (RIP)

To determine the interaction of RNAs and proteins, an RIP assay was conducted in the light of the manufacturer’s protocol of the Magna RIP™ RNA-Binding Protein Immunoprecipitation Kit (Cat#17–700, Millipore, Germany). RNAs combined with protein were isolated and then detected by qRT-PCR.

### RNA pull-down

Full-length FIRRE and antisense sequences were synthesized and purified as above. Then the RNA transcripts were labeled by Desthiobiotin at 16 °C overnight (Cat#20163, Thermo Scientific, USA). RNA pull-down assays were performed using the Magnetic RNA-Protein Pull-Down Kit (Cat#20164, Thermo Scientific, USA) according to the manufacturer’s instructions. The retrieved protein was detected with a silver staining kit (Thermo) or by standard immunoblotting.

### Sliver stain assay

Proteins in polyacrylamide gels was stained using Pierce™ Silver Stain for Mass Spectrometry kit (Cat#24600, Thermo, USA). Procedures for protein staining were carried out following the instructions.

### Subcellular fractionation

Cells with 90–100% confluence were harvested and washed twice with cold PBS. The cytoplasmic and nuclear protein extraction was performed according to the manuscript of Minute^TM^ Cytoplasmic and Nuclear Extraction Kit for Cells (Cat#SC-003, Invent, USA). Appropriate amounts of cytoplasmic extraction buffer were added to the cells and incubated on ice for 5 min. The lysate was centrifuged for 5 min at top speed in a microcentrifuge at 4 °C and supernatant (cytosol fraction) was transferred to a fresh pre-chilled 1.5 ml tube. Appropriate amounts of nuclear extraction buffer were added to the pellet. After vortexing and incubating five times, the lysate was centrifuged at top speed (14,000–16,000 × *g*) in a microcentrifuge for 30 s. LaminB1 (Cat#13435, CST, USA) and GAPDH (Cat#60004-1-Ig, Proteintech, USA) serves as the nuclear control and cytosolic controls, respectively.

### Protein extraction and western blotting

Cells were lysed in RIPA buffer (Cat#KGP702, KeyGene BioTECH, China) supplemented with protease and phosphatase followed by brief sonication. Protein concentrations were measured by KeyGene BioTECH kit (Cat#KGP902, KeyGene BioTECH, China) according to instructions. After heat denaturation, the lysed proteins were resolved on SDS-PAGE gels and transferred onto a PVDF membrane (Cat#IPVH00010, Millipore, USA). After being incubated in 5% nonfat milk (Cat#P0216, Beyotime Biotechnology, China) dissolved in TBS-Tween 20 (0.1%; Cat#ST825, Beyotime Biotechnology, China) solution for 1 h, the membrane was incubated with primary antibodies overnight at 4 °C. After being washed three times, the membrane was incubated in the HRP-conjugated secondary antibody. The signals were captured using a Bio-Rad ChemiDoc MP System.

### Fluorescence in situ hybridization

RNA in situ hybridization using FIRRE RNA probes was performed according to the manufacturer’s instructions. Three biotin-labeled RNA probes were synthesized by GenePharma (Shanghai, China), and the confocal microscope was utilized to detect the RNA-FISH data. Sequences of RNA probes for FIRRE FISH are listed in Supplementary Table 2.

### Immunofluorescence

Cells were fixed with 4% paraformaldehyde (Cat#KGIHC007, KeyGEN BioTECH, China) after washing by PBS. Subsequently, the cells were permeabilized by the TritonX-100 (Cat# Beyotime Biotechnology, China), then washed and treated with blocking buffer for 1 h at RT. The cells were incubated with anti-PTBP1 antibody at 4 °C overnight. On the next morning, the secondary antibody was incubated after washing three times with PBST according to the manufacturer’s protocol. After incubated with DAPI (KeyGene BioTECH, China) for 15 min at RT, the cells were analyzed through a microscope.

### RNA stability assay

Expression of PTBP1 and FIRRE were knocked down alone or in combination in RKO and HCT116 cells. Actinomycin D (ACD) (MedChemExpress, China) (5 µg/ml) was used to block *de novo* transcription in the cells above. After treatment with ACD for 60 min, mRNA level was measured by qPCR.

### LncRNA-seq and sequencing analysis

Three pairs of tissues were tested for lncRNA RNA-sequencing profile analysis. RNAs were extracted from the colorectal cancer tissues using TRIzol (Cat#15596018, Invitrogen, USA) and 2 μg of total RNAs for each sample were used to prepare the lncRNA-seq library according to the manufacturer’s instruction of the Illumina TruSeqTM RNA Sample Prep Kit. Then, the cDNAs were sequenced by Illumina HiSeq platform according to the manufacturer’s instructions.

### Analysis of RNA-seq

The reads of each sample were mapped against the human genome(http://asia.ensembl.org/Homo_sapiens/Info/Index) through TopHat. Cuffdiff was used to quantify differentially expressed genes/transcripts. We restricted analysis to genes significantly differentially expressed by at least |log2FC | > 1 and attached *P* < 0.05 and FDR < 0.01 to statistical significance.

### Bioinformatic analysis

In order to explore the effect of FIRRE on patients with CRC, the bioinformatic analysis was conducted via R package. The data were acquired from TCGA, containing total of 435 patients with colon adenocarcinoma.

### Rapid prediction of the interaction between RNA and protein

To further investigate the mechanism of FIRRE in carcinogenesis, potential proteins interact with RNAs were evaluated using catRAPID. catRAPID was used to measure the interaction probability between protein (or region) and RNA (or region). The discriminative power (DP), ranging from 0% to 100%, is a statistical measure to evaluate the interaction propensity. DP values above 50% means that the interaction is likely to occur, while DP above 75% indicates high-confidence predictions [24]. All predictions would be tasted again using starbase2.0 (http://starbase.sysu.edu.cn/starbase2/index.php).

### Statistics analysis

Data are expressed as mean ± SD. Analyses were performed using Graph Pad Prism software. Group comparisons for continuous data were compared by Student *t*-test. One-way ANOVA was performed when groups were >3. Kaplan–Meier method and Log-rank analysis were used for survival analysis. For each test, differences between the values were considered statistically significant when *p* < 0.05.

## Results

### The expression of FIRRE is upregulated in colorectal cancer samples

**Fig. 1:**
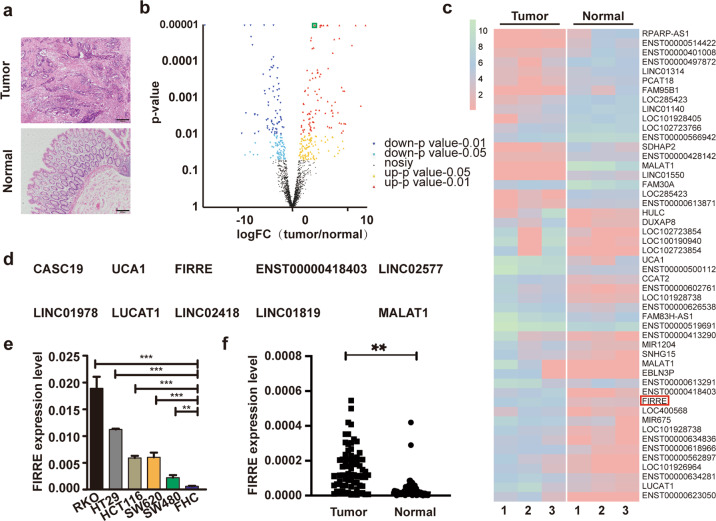
The expression of FIRRE in colorectal cancer samples. **a** HE stain was used to verify the cancer and normal tissues. **b** Volcano-plots was used to show the differential lncRNAs in samples. The *x*-coordinate means the fold change of expression difference between two samples. The ordinate shows the statistical value. The dot refers to one gene. Red dots represent genes that are significantly upregulated. Blue dots represent genes that are significantly downregulated. Black dots represent genes that are not significantly different. FIRRE is marked by green box. **c** The heatmap depicts part of the differential lncRNAs, and FIRRE is marked by red box. **d** According to the value of FDR, the top 10 upregulated genes in cancer tissues comparing with normal ones were listed. **e** Expression level of FIRRE was quantitated by qRT-PCR in colon carcinoma cell lines, including RKO, HCT29, HCT116, SW620, and SW480, and in normal intestinal epithelial cell line FHC. **f** qRT-PCR was conducted to test the expression of FIRRE in cancer tissues and normal tissues. Data are shown in means ± SD from triplicate assays. Bars, ±SD; Statistical analysis: ANOVA test, *P < 0.05, **P < 0.01.*p < 0.05, **p < 0.01, ***p < 0.0001.

The cancerous and normal tissues were distinguished through HE staining (Fig. 1a). Differential lncRNA expression in paired tissues of tumor and adjacent normal tissues was evaluated by high throughput sequencing. Volcano plot (| log2FC | > 1, *p*-value < 0.05) (Fig. 1b) revealed the overall gene expression levels. Among which, FIRRE was overexpressed in cancer tissues. The results were also demonstrated in heatmap (Fig. 1c). Combining log2| FC | > 1, the top ten upregulated genes in cancer tissues were listed according to the value of FDR (Fig. 1d). Results of qPCR in CRC cell lines showed that FIRRE was overexpressed in cancer cells (Fig. 1e). The overexpression of FIRRE was also detected in tissues from 74 patients with CRC (Fig. 1f). These findings revealed that FIRRE is upregulated in CRC.

### FIRRE promotes CRC cells migration, invasion, and proliferation

**Fig. 2:**
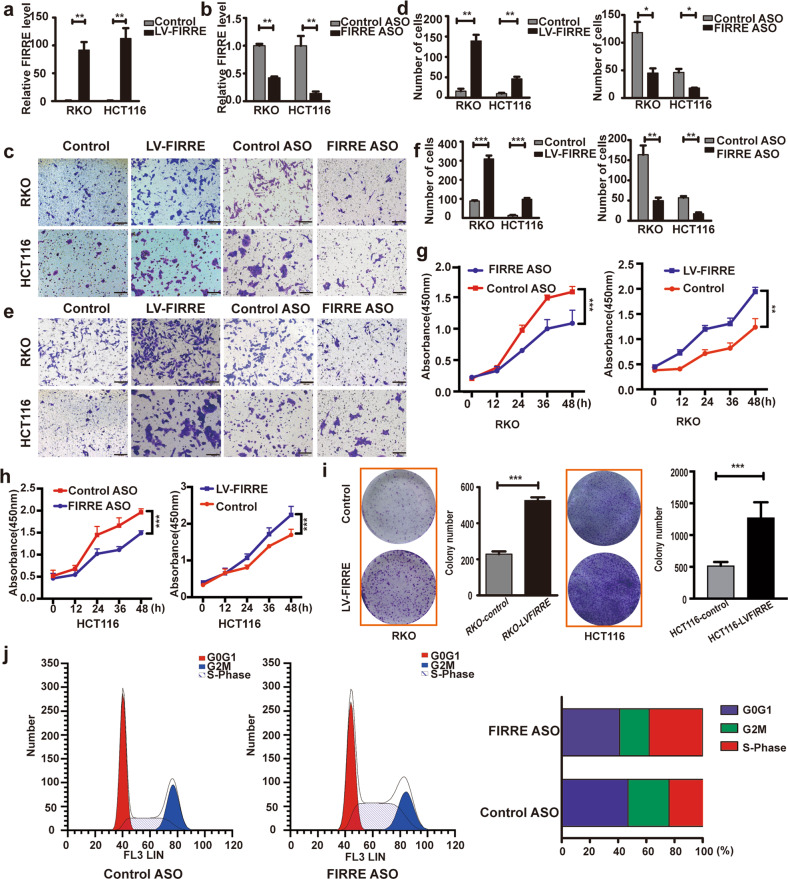
FIRRE promotes CRC cells migration, invasion, and proliferation. **a** qPCR analysis was used to test level of FIRRE in RKO and HCT116 cells transfected with control vector or LV-FIRRE. **b** The level of FIRRE in RKO and HCT116 cells with Control ASO or FIRRE ASO. **c**, **d** The migration assay to explore migration ability of cells with LV-FIRRE or FIRRR ASO. Photos were taken under inverse microscope, Scale bar = 100 µm. *p < 0.05, **p < 0.01. **e**, **f** The invasion assay to test the invasion ability of cells with LV-FIRRE or FIRRR ASO, Scale bar = 100 µm. **g** CCK-8 assays were performed to investigate proliferation of RKO cells with LV-FIRRE or FIRRE ASO as above. Data are presented as mean ± SD for four wells and are representative of three separate times. **h** CCK-8 assays were performed to investigate proliferation of HCT116 cells with LV-FIRRE or FIRRE ASO. Data are presented as mean ± SD for four wells and are representative of three separate times. **i** Colony formation assays were utilized to test the colony formation ability in RKO and HCT116 cells after FIRRE overexpression. Data are shown means ± SD in three repeated experiments. **j** Flow cytometry showed that the S-phase arrest was observed after FIRRE knockdown. Bars, ±SD; Statistical analysis: *t*-tests *p < 0.05, **p < 0.01, ***p < 0.0001.

Overexpression of FIRRE in CRC suggested that FIRRE may play roles in CRC development. To verify the speculation, LV-FIRRE and ASO-FIRRE were transfected in RKO and HCT116 cells, respectively. Expression of FIRRE was effectively changed in transfected cell lines (Fig. 2a, b). Transwell assay revealed that the FIRRE depletion significantly inhibited the invasion and migration of colorectal cancer cells (Fig. 2c, d). On the contrary, FIRRE overexpression increased invasion and migration ability (Fig. 2e, f). Proliferation assay showed overexpression of FIRRE significantly promotes cell growth, while FIRRE silencing could significantly inhibit cell growth (Fig. 2g, h). Colony formation assays showed that colony numbers in cells with LV-FIRRE were more abundant than that of control (Fig. 2i). Flow cytometry demonstrated that silencing of FIRRE induced significant S-phase arrest (Fig. 2j). These findings indicated that FIRRE has a promotive effect on cell proliferation, invasion, and migration.

### FIRRE overexpression is related to CRC progression in patients

**Fig. 3:**
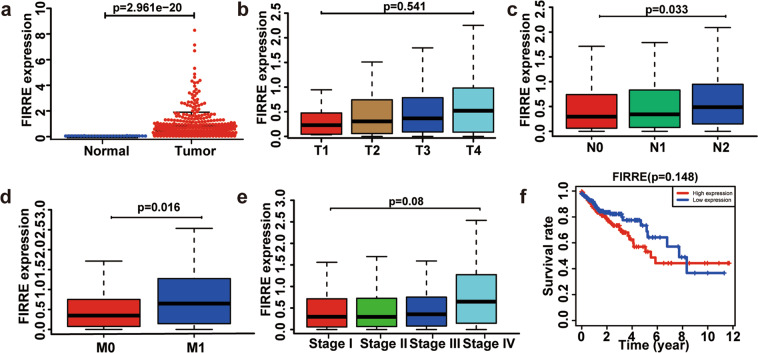
FIRRE functions as an oncogenic factor in CRC patients. **a** The level of FIRRE in CRC patients. *p*-value is indicated in the plot. **b** The data show that level of FIRRE was not related with T staging in CRC patients. *p*-value = 0.541. **c** The figure shows that higher FIRRE was associated with more advanced N staging. *p*-value = 0.033. **d** The figure shows that higher FIRRE was associated with more advanced M staging. *p*-value = 0.016. **e** The levels of FIRRE are not associated with the overall TNM staging. *p*-values are shown in the plot. **f** FIRRE makes no difference in the Overall Survival of patients with colorectal cancer. Bars, ±SD; Statistical analysis: ANOVA test, *t*-tests.

In order to investigate the prognostic value of FIRRE, we performed bioinformatic analysis. The characteristic of patients from TCGA data was shown in Supplement Table 3. Results indicated that FIRRE was significantly upregulated in CRC (Fig. 3a). The level of FIRRE was positively related to lymphatic and distant metastasis, rather than depth of invasion (Fig. 3b, c, d). Although increased FIRRE was associated with a lower survival rate, there was no statistical significance (Fig. 3f). Moreover, the level of FIRRE has nothing to do with sex and age (Supplement Fig. 1)

The characteristics of patients with CRC was shown in Table 1. As summarized in Table 1, FIRRE was positively related to the advanced TNM stage. Patients with higher FIRRE levels suffered a higher incidence of lymphatic and distant metastasis (*p* < 0.05). FIRRE was not related to sex, age, and tumor size (*p* > 0.05), which was consistent with the results of bioinformatic analysis. Taken together, our results indicated that FIRRE positively correlated with CRC progression.

**Table 1:** Correlations between FIRRE expression and CRC patient clinicopathologic features.

*FIRRE* expression				
	No. (%)	High	low	*p*-value
*Sex*				0.684
Male	39 (52.70)	17 (43.59)	22 (56.41)	
Female	35 (47.30)	18 (51.42)	17 (48.57)	
*Age (years)*				0.252
<60	33 (44.59)	15 (45.45)	18 (54.54)	
≥60	41 (55.41)	19 (46.34)	22 (53.66)	
*Location*				0.807
Colon	49 (66.22)	24 (48.98)	25 (51.02)	
Rectum	25 (33.78)	11 (44.00)	14 (56.00)	
*Tumor size (cm)*				0.117
<3 cm	26 (35.14)	14 (53.85)	12 (46.15)	
≥3 cm	48 (64.86)	26 (54.17)	22 (45.83)	
*Stage*				<0.001
I/II	32 (43.24)	21 (65.62)	11 (34.38)	
III/IV	42 (56.75)	34 (80.95)	8 (19.05)	
*Lymphatic metastasis*				<0.001
No	34 (45.95)	18 (52.94)	16 (47.06)	
Yes	40 (54.05)	34 (85.00)	6 (15.00)	
*Distant metastasis*				<0.001
No	52 (70.27)	35 (67.31)	17 (26.92)	
Yes	22 (29.73)	21 (95.45)	1 (4.55)	

### FIRRE directly interacts with RNA-bind protein PTBP1

**Fig. 4:**
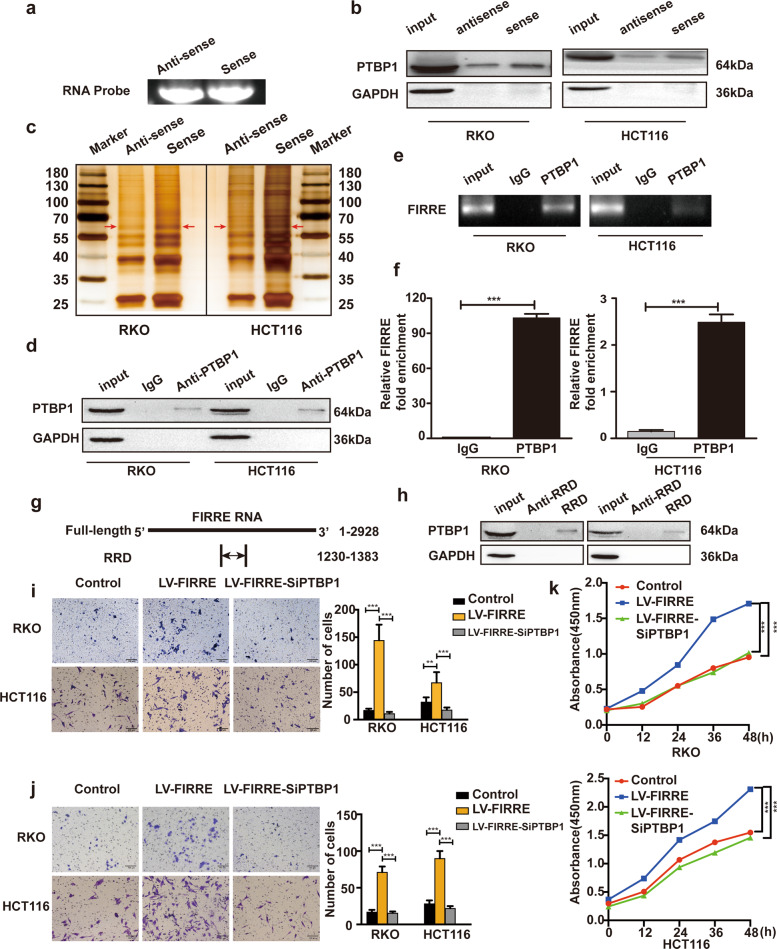
FIRRE directly interacts with RNA-binding protein PTBP1. **a** The antisense and sense of FIRRE were synthesized in vitro through RiboMAX™ Large Scale RNA Production Systems–SP6 and T7 (Promega). **b** RNA pull-down was conducted with the antisense or sense of FIRRE. Western blot with PTBP1 antibody shows the sense of FIRRE makes more PTBP1 enrichment compared with the antisense. **c** The silver stain further identified the binding between PTBP1 and FIRRE. PRBP1 was marked with the red arrows. **d** PTBP1 RIP assay to analyze interactions between PTBP1 and FIRRE in both RKO and HCT116 cells. WB shows the PTBP1 antibody efficiency of immunoprecipitation. **e** RT-PCR using RIP Primers specific for the FIRRE to validate the interaction between FIRRE and PTBP1. PCR product was observed in the anti-PTBP1 RIP (lane 3) and substantially less was detected in the normal IgG RIP (lane 2). **f** qRT-PCR to analyze the enrichment of FIRRE in RNA-protein complexes. The FIRRE abundance in anti-PTBP1 group was much more than the IgG group, normalized by the input group. **g** RRD was synthesized in vitro. RRD was presumed as the binding sites of FIRRE RNA to the PTBP1 protein. **h** RNA pull-down was conducted with the antisense or sense of RRD. Western blot with PTBP1 antibody shows the sense of RRD makes more PTBP1 enrichment compared with the antisense. **i** The migration assay to explore the migration ability of cells with FIRRE overexpression and cells with LV-FIRRE and siPTBP1. Photos were taken under inverse microscope, Scale bar = 100 µm. **j** The invasion assay to test the invasion ability of cells with FIRRE overexpression and cells with LV-FIRRE and siPTBP1, Scale bar = 100 µm. **k** CCK-8 assays were performed to investigate proliferation of cells with LV-FIRRE or LV-FIRRE and siPTBP1. All experiments were repeated double times or more. Bars, ±SD; Statistical analysis: ANOVA test, *t*-tests. **p < 0.01, ***p < 0.0001.

It has been reported that FIRRE interacts with hnRNP U [11]. PTBP1, member of hnRNP family, was predicted to bind FIRRE with its DP value above 75% using catRAPID, which was evaluated again using Starbase2.0. We conducted RNA pull-down and RIP assay to verify the direct physical interaction. FIRRE RNA probes (sense and antisense of FIRRE) were synthesized through in vitro transcription (Fig. 4a). Subsequently, PTBP1 in RNA-protein complexes were tested through SDS-PAGE (Fig. 4b) and silver staining assay (Fig. 4c). The results exhibited that the sense of FIRRE but not antisense captured a distinct band of PTBP1. For RIP assay, western blotting was firstly conducted to test the efficiency of immunoprecipitation (Fig. 4d). The purified immunoprecipitated RNA was then analyzed by RT-PCR and qPCR. Compared with the normal anti-IgG, the anti-PTBP1 enriched more FIRRE RNA (Fig. 4e, f). Repeating RNA Domain (RRD) is the only repeat located in the exons of the FIRRE transcript and is proven to physically interact with hnRNP U [11]. suggesting that FIRRE may bind PTBP1 in an RRD-dependent manner. The RRD probes (antisense and sense) were prepared using in vitro transcription (Fig. 4g). Results of RNA pull-down showed that PTBP1 preferentially co-precipitated with RRD domain of FIRRE (Fig. 4h). These results provided direct evidence of interaction between PTBP1 protein and FIRRE RNA, indicating the interaction may be involved in the oncogenic role of FIRRE in CRC.

### The function of FIRRE in CRC was influenced by PTBP1

To investigate the roles of PTBP1 in FIRRE-mediated oncogenic function in CRC, we knocked down PTBP1 in cells with LV-FIRRE. The efficiency of PTBP1 silence at RNA and protein level was shown in Supplement Fig. 2. Transwell assay revealed that the PTBP1 depletion significantly reduced the invasion and migration ability in cells with LV-FIRRE (Fig. 4I, j). Proliferation assay showed silence of PTBP1 could significantly inhibit cell growth compared with cells with LV-FIRRE (Fig. 4k).

### The interaction between PTBP1 and FIRRE changes the subcellular localization of PTBP1 and FIRRE

**Fig. 5:**
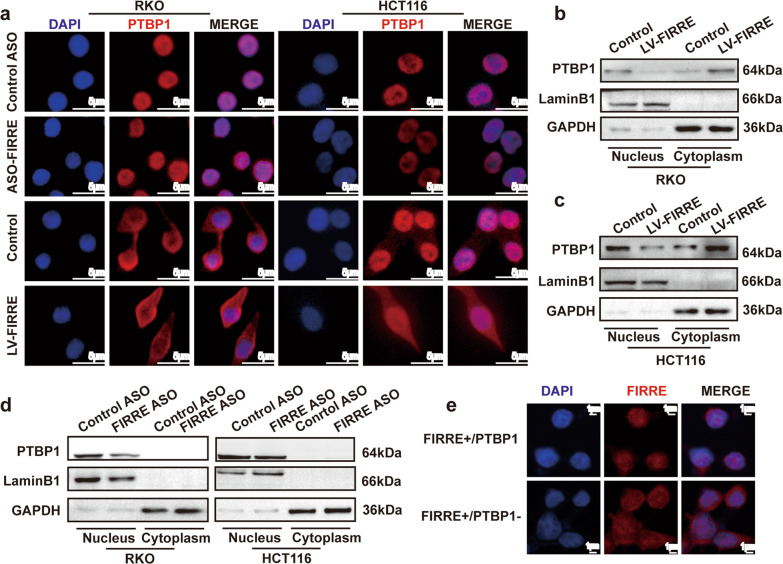
FIRRE leads to the translocation of PTBP1. **a** Immunofluorescence to test the subcellular localization of PTBP1 in cells transfected with FIRRE ASO or lentivirus FIRRE. The picture shows the blue DAPI fluorescence of nuclei and the red staining of anti-PTBP1 antibodies with Cy3-labeled secondary antibodies. **b**, **c**, and **d** Western blot assay of cytosol and nuclear proteins were used to verify the subcellular location of PTBP1 in RKO and HCT116 cells with differential level of FIRRE. GAPDH acted as the marker of cytoplasm, and LaminB1 as the marker of nuclear. b and c, RKO and HCT116 cells with transfection of control vector or lentivirus FIRRE. **d** RKO and HCT116 cells with Control ASO or FIRRE ASO. **e** FIRRE localization was determined by RNA-FISH. RKO cells with high expression of FIRRE were transfected with control siRNA (up) or PTBP1 siRNA (down). The nuclear was labeled by DAPI (bule) and the FIRRE RNA probe was stained by Cy3 (red).

It was reported that PTBP1 was able to shuttle between nucleus and cytoplasm [25]. Immunofluorescence was conducted to explore the effect of FIRRE expression on subcellular localization of PTBP1 protein. PTBP1 protein and nucleus were stained by Cy3-labeled secondary antibody and DAPI, respectively. Immunofluorescence microscopy revealed that PTBP1 protein was restricted to the nucleus after FIRRE depletion; while PTBP1 protein was present in the cytoplasm after FIRRE overexpression (Fig. 5a), indicating levels of FIRRE influenced the localization of PTBP1.The cytosol and nuclear proteins were separated, and then WB assay was applied to further identify the subcellular localization of PTBP1. Being consistent with results of immunofluorescence, FIRRE facilitated the shuttle of PTBP1, promoting PTBP1 translocation from nucleus to cytoplasm (Fig. 5b, c, d).

Interestingly, level of PTBP1, in turn, also affected the localization of FIRRE. PTBP1 expression was downregulated using siRNA in cells with FIRRE overexpression. Subsequently, FISH targeting FIRRE was performed to observe the location of FIRRE. A distinct delocalization and translocation of FIRRE from nuclear to the cytoplasm was observed in cells with PTBP1 silence (Fig. 5e). Results above suggested that the subcellular distribution was changed due to the interaction of FIRRE and PTBP1.

### BECN1 mRNA is the target mRNA of PTBP1 protein

**Fig. 6:**
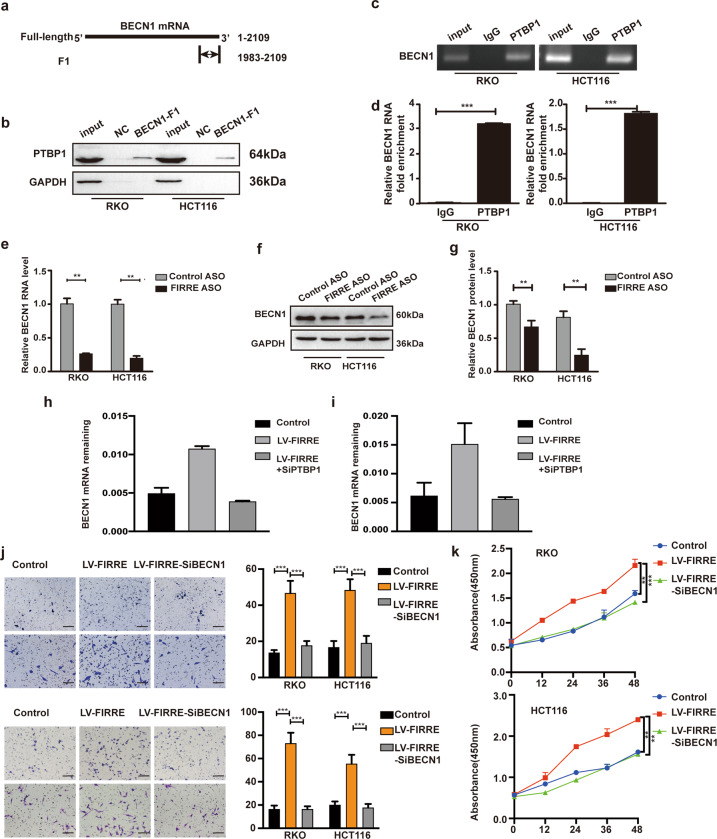
The RNA-protein complex interacts with and stabilizes BECN1 mRNA. **a** Fragment of BECN1 mRNA (named F1) was synthesized in vitro. F1 was presumed as the binding sites of BECN1 mRNA to the PTBP1 protein. **b** RNA pull-down with F1 RNA probe and control RNA probe was used to identify the interaction of BECN1 and PTBP1. Western blot with PTBP1 antibody shows F1 enriches more PTBP1 compared with the control. **c** RNA immunoprecipitation (RIP) of PTBP1 interaction with BECN1 RNA in vivo in RKO and HCT116 cells. Protein-RNA complexes captured by anti-PTBP1 or IgG were determined by RT-PCR using specific primers. PCR product was observed in the anti-PTBP1 RIP and substantially less was detected in the normal IgG. **d** qRT-PCR to analyze the enrichment of FIRRE in RNA-protein complexes. The FIRRE abundance in anti-PTBP1 group was much more than the IgG group, normalized by the input group. **e** FIRRE depletion induces the decrease of BECN1 mRNA level, which was demonstrated by qRT-PCR. **f**, **g** Western blot assay was further used to verify the influence of FIRRE silence on BECN1 mRNA stability in RKO and HCT116 cells. **g** Quantification of the western blot. **h**, **i** RKO(h) and HCT116(i) cells transfected with siPTBP1 or LV-FIRRE alone or in combination were treated with actinomycin D (5 μg/ml). RNA was extracted at 60 mins, and qPCR was used to quantify BECN1 mRNA. All experiments were repeated double times or more. *p < 0.01; **p < 0.001; ***p < 0.0001. **j** The migration and invasion assay to explore the migration ability of cells with FIRRE overexpression and cells with LV-FIRRE and siBECN1. Photos were taken under inverse microscope, Scale bar = 100 µm. **k** CCK-8 assays were performed to investigate proliferation of cells with LV-FIRRE or LV-FIRRE and siBECN1. All experiments were repeated double times or more. Bars, ±SD; Statistical analysis: ANOVA test, *t*-tests. **p < 0.01, ***p < 0.0001.

The cellular functions of PTBP1 are dependent on its location. In cytoplasm, PTBP1 regulates mRNA metabolism via binding target mRNAs. The interaction between PTBP1 and FIRRE changed the subcellular localization of PTBP1. Thus, it was postulated that the association between FIRRE and PTBP1 may have impact on target mRNAs of PTBP1. BECN1 was predicted as the target mRNA of PTBP1 using catRAPID and contains potential PTBP1 binding sites in its 3′UTR (Fig. 6a). To identify this hypothesis, the fragment of BECN1 mRNA (BECN1-F1) was synthesized through in vitro transcription and the quality was tested by Sanger sequencing.

RNA pull-down showed BECN1-F1(from 1983 to 2109), instead of the control, can successfully enrich PTBP1 protein (Fig. 6b). Subsequently, RIP assay was implemented with anti-PTBP1 and lgG antibodies. Consistent with result of RNA pull-downs, RIP assay indicated that stronger enrichment of BECN1 mRNA was observed in anti-PTBP1 group compared with IgG controls after normalization to total input (Fig. 6c, d). Overall, the results suggested the BECN1 mRNA is target mRNA of PTBP1 protein.

### FIRRE enhances BECN1 mRNA stability in PTBP1-mediated manner

Based on the function of PTBP1 in cytoplasm on mRNA stability [25], we hypothesized that FIRRE and PTBP1 could modulate BECN1 mRNA stability. To validate this hypothesis, the mRNA and protein levels of BECN1 were first tested in cells with ASO-FIRRE and the control. It was shown that BECN1 expression at mRNA and protein level was remarkably decreased after FIRRE downregulation (Fig. 6e, f, g). The cells with LV-FIRRE or LV-FIRRE and SiBECN1 were treated by Actinomycin D in order to block de novo transcription. qPCR results revealed that FIRRE overexpression increased the stability of BECN1 mRNA; however, PTBP1 depletion in cells with LV-FIRRE increased the degradation of BECN1 mRNA. (Fig. 6h, i). We knocked down FIRRE and PTBP1 alone or in combination in cells and then treated reprogrammed cells with Actinomycin D. Our results revealed that depletion of FIRRE or PTBP1 increased the degradation of BECN1 mRNA; Under the condition of co-depletion of FIRRE and PTBP1, BECN1 mRNA stability was reinforced (Supplement Fig. S3a, b). These results indicated that the FIRRE stabilized BECN1 mRNA stability and PTBP1 played important roles in FIRRE-mediated RNA stability.

### BECN1 is indispensable for FIRRE-mediated progress of CRC

Since the function of FIRRE in BECN1 mRNA stability, we investigated whether BECN1 mediates the regulation of FIRRE on CRC. Transwell assay revealed that the BECN1 depletion significantly reduced the invasion and migration ability in cells with LV-FIRRE (Fig. 6j). Proliferation assay showed silence of PTBP1 could significantly inhibit cell growth compared with cells with LV-FIRRE (Fig. 6k).

### FIRRE contributes to autophagy activity

**Fig. 7:**
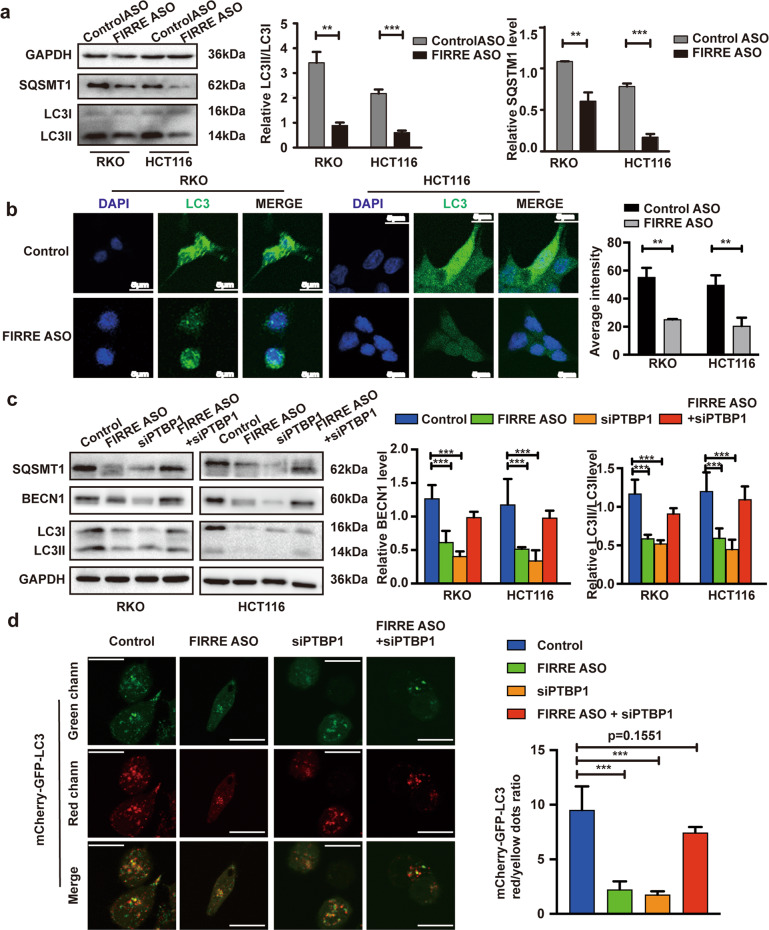
FIRRE activates autophagy. **a** Western blots of important autophagy-associated proteins, including SQSMT1 and LC3, in RKO and HCT116 with FIRRE knock-down. Statistical analysis of the WB of SQSMT1 and LC3 is on the right. **b** The photomicrographs of RKO and HCT116 cells transfected with ASO-FIRRE and stained by indirect immunofluorescence for nuclear (DAPI, blue), and LC3 protein (labeled by Alexa 488, green). Statistical analysis of the average intensity is on the right. **c** Expression level of BECN1, SQSMT1, and LC3 were detected in cells transfected with siPTBP1 or ASO-FIRRE alone or in combination. **d** Cells with siPTBP1 or ASO-FIRRE alone or in combination were transfected with lentivirus mCherry-GFP-LC3 to observe the autophagy flux. Then, the presence of GFP-mCherry- (autophagosomes, yellow) and mCherry-positive (autolysosomes, red) dots were obsserved using confocal microscopy. Scale bar = 5 µm. Right panel: quantification of the ratio between red vs. yellow dots to assess an autophagy flux index. All experiments were repeated double times or more. Bars, ±SD; Statistical analysis: ANOVA test, *t*-tests. *p < 0.01; **p < 0.001; ***p < 0.0001.

Considered the important role of BENC1 in autophagy, we detected levels of p62 and LC3. Western blot demonstrated lower enrichment of p62 and LC3 in cells with FIRRE depletion compared with the controls (Fig. 7a), indicating the reduction of autophagosome formation. In addition, immunofluorescence showed that the autophagic compartments in cells with FIRRE depletion were decreased, indicating a diminution in autophagy (Fig. 7b). These results suggested FIRRE sustains autophagy activity. To further investigate the roles of PTBP1 in FIRRE-mediated autophagy activity, we evaluated the expression of BECN1, p62, and LC3 in cells with siPTBP1 or ASO-FIRRE alone or in combination. Results of western blot revealed that the depletion of FIRRE or PTBP1 decreased the levels of BECN1, p62, and LC3. Under the condition of co-depletion of FIRRE and PTBP1, levels of BECN1, p62, and LC3 were revised (Fig. 7c). Finally, we confirmed the inhibition of autophagy flux by FIRRE and PTBP1 depletion in RKO cells using the mCherry-GFP-LC3 reporter (Fig. 7d). Together, these results indicate that FIRRE induces the activity of autophagy.

## Disscussion

Overwhelming evidence indicates that LncRNAs contribute to numerous human diseases, especially in cancers [26]. Although previous research has well-revealed functions of FIRRE in other diseases, little is known about the function and mechanism of FIRRE in cancers. In the present study, lncRNA FIRRE is confirmed to function as an oncogenic factor in CRC. Our data show that FIRRE has extraordinarily higher expression in tumor samples than in normal ones, which is consistent with previous study from Meng Li [27]. Overexpression of FIRRE promotes tumor cell proliferation, migration and invasion. Results of the cell cycle demonstrate that FIRRE silencing causes a significant S-phase arrest. S-phase is a stage that determines DNA syntheses. The cells were arrested in S-phase, and fewer cells were in cell division phase. Accompanying the decrease of G2/M, S-phase accumulation indicates that FIRRE silencing inhibits cell division. These results suggest that FIRRE may facilitate the cell division. The *in silico* analysis also indicates that FIRRE is associated with lymphatic and distant metastasis. In agreement with the results from *in silico* analysis, CRC patients with higher expression suffer from a more advanced disease staging than those with lower FIRRE level. The roles of FIRRE in patients with CRC are consistent with the results in vitro, indicating FIRRE is positively associated with the development and progression of CRC.

The subcellular localization of lncRNAs can often provide clues to their own function [28]. LncRNAs are predominantly located in the nucleus [29]. The nuclear lncRNA functions primarily by interaction with proteins, RNA, or genomic DNAs [15]. FIRRE is nuclear-localized and has been reported that FIRRE binds hnRNP U to maintain or establish higher-order nuclear architecture [11]. According to the above, we selected PTBP1 as target protein among the proteins which were predicted to bind with FIRRE. We further validated the physical interaction between FIRRE RNA and the PTBP1 protein using RNA pull-down and RIP analysis. The interactions of lncRNAs and hnRNPs are pervasively involved in modulating cell functions at transcriptional and post-transcriptional levels. For instance, LincRNA-p21 forms a complex with hnRNP K to activate p21 transcription [30]. LincRNA-Cox2 repress genes that are related to the immune response expression with HnRNP A/B as lincRNA-Cox2 binding partner [31]. LncRNAs and hnRNPs interaction contributes to mRNA stability and translation at post-transcriptional levels. Linc-RoR aggravates oncogenesis by enhancing c-Myc mRNA and protein levels, which is resulted from binding hnRNP I [8]. The effect of interaction between PTBP1 and FIRRE was further investigated in our research.

Combining the alterable localization of PTBP1 in nucleus and cytoplasm, our results demonstrate that the interaction of FIRRE and PTBP1 contributes to the translocation of PTBP1. FIRRE overexpression induces the cytoplastic localization of PTBP1. It is known that PTBP1 contains a nuclear localization sequence (NLS) [32] and four RNA recognition motifs (RRMs) [33]. FIRRE may bind the RRMs of PTBP1. NLS alone is not enough to maintain the steady nuclear localization of PTBP1, but instead, the joint effect of the NLS and RRMs constitute the nucleus-located domain. Interaction of FIRRE and PTBP1 via RRMs may weaken the combined effect of nuclear localization, resulting in the translocation of PTBP1 protein. The shuttling of hnRNPs, including PTBP1, is closely coupled with RNA export. Our results also verify that the delocalization of FIRRE is observed after PTBP1 silencing. Repeating RNA Domain (RRD), a conserved domain in FIRRE, is the potential protein binding site and necessary for the proper localization of FIRRE [11]. Through results from RNA pull-down using RRD fragment, we confirmed that FIRRE binds PTBP1 via RRD. These suggest the RRD is necessary for the proper localization of FIRRE, which is consistent with the study of Hacisuleyman et al. [11].

Shuttling hnRNPs are implicated in both nuclear and cytoplasmic functions. Function of PTBP1 depend on its cell localization [17]. When localizing in cytoplasm, PTBP1 regulates the splicing, polyadenylation, mRNA stability, and translation initiation in a way of interacting with its target mRNA [34]. These remind us that FIRRE and PTBP1 may work through interacting with target mRNAs of PTBP1. BECN1 mRNA is predicted to be enriched by PTBP1. The prediction is confirmed by RNA pull-down using 3'-UTR of BECN1 mRNA. It is further verified via the RIP experiment with PTBP1 antibody. These results indicate the formation that BECN1 was the target mRNA of PTBP1 protein.

Considerable researchers have concluded that the combination of lncRNAs and hnRNPs controls mRNA stability. Linc-RoR stabilizes c-Myc mRNA by interacting with hnRNP I and AUF1 [8]. LncRNA UCA1 physically interacts with PTBP1 and stabilizes ALAS2 mRNA [35]. These studies suggest that interaction of FIRRE and PTBP1 may affect the stability of BECN1 mRNA. Results of qPCR indicate that FIRRE overexpression sustains a high level of BECN1 mRNA while PTBP1 depletion in cells with LV-FIRRE impairs BECN1 mRNA stability. These results support that PTBP1 plays important role in FIRRE-mediated mRNA stability. however, FIRRE or PTBP1 depletion alone decreases BECN1 mRNA stability while loss of both FIRRE and PTBP1 reverses the results above. The relationship between FIRRE and PTBP1 is complicated, which requires further investigation.

We further investigated the roles of BECN1 in FIRRE-mediated carcinogenic function in CRC. Our results demonstrated BECN1 is indispensable for FIRRE-mediated progress of CRC. The loss of BECN1 weakened the tumor-promoting effect of FIRRE in CRC. Given the role of BECN1 in autophagy, FIRRE was deduced to contribute to autophagy activity. Our results showed the promotive function of FIRRE in autophagy. Loss of FIRRE contributes to the decreased level of crucial members in autophagy, such as p62 and LC3II. We also monitored autophagic fluxes in the absence of FIRRE or/and PTBP1. Knockdown of FIRRE inhibits the fusion of autophagosomes with lysosomes and induces blockage of autophagic fluxes. Function of autophagy in cancers is controversial. Autophagy may have a significant role in inhibiting tumorigenesis at the earliest stages; however, in established cancers, autophagic responses may favor tumor progression through helping to deal with intracellular and environmental stress [36, 37]. Our results suggested that FIRRE promotes CRC progression through increasing activity of autophagy in advanced CRC. However, the inhibition of autophagy was reversed when knocking down FIRRE and PTBP1 in combination. The roles of PTBP1 in autophagy are little known. PTBP1 was reported to downregulate ATG10 mRNA level [38], suggesting the inhibitory effect on autophagy. Han et al reported that ZNF649-AS1 activates the transcription of ATG5 by recruiting PTBP1 [39], indicating the stimulative effect on autophagy. In this study, depletion of PTBP1 decreases the level of p62 and LC3, and induces the blockage of autophagic fluxes. These results demonstrate that PTBP1 functions as a promotive factor on autophagy.

**Fig. 8:**
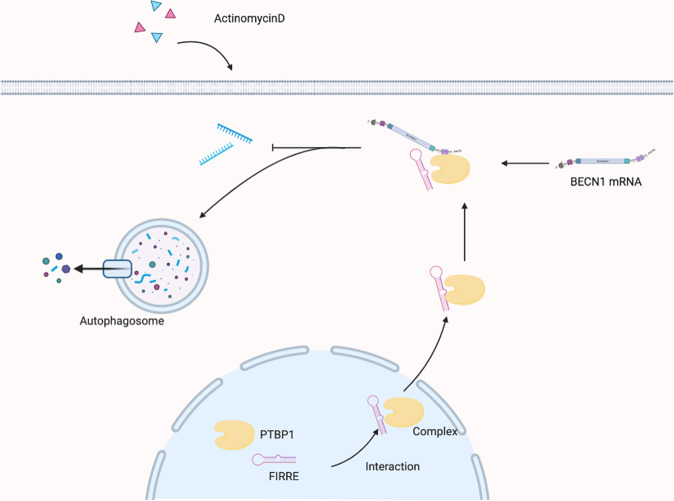
The mechanism of FIRRE in CRC progress. FIRRE which is localized in the nucleus binds to PTBP1 protein, promoting the translocation of PTBP1 from nucleus to cytoplasm. The RNA-protein complex in cytoplasm interacts with the 3' end of BECN1 mRNA. This interaction enhances the stability of BECN1 mRNA under treatment of ACD and the activity of autophagy, which contributes to the development of CRC.

In summary, our study provides evidence that lncRNA FIRRE promotes CRC development and progression. Mechanistic analyses revealed that FIRRE stabilizes BECN1 mRNA and contributes to autophagy (Fig. 8). This study sheds new light on the function and mechanism of FIRRE in CRC.

## Supplementary information

Supplement

FigS1

FIG S2

Fig S3

Related Manuscript File

Related Manuscript File

Related Manuscript File

Related Manuscript File

Related Manuscript File

Related Manuscript File

Related Manuscript File

Related Manuscript File

## Data Availability

All data generated or analyzed during this study are included in this published article and its supplementary information files.

## References

[1] Siegel RL, Miller KD, Goding Sauer A, Fedewa SA, Butterly LF, Anderson JC (2020). Colorectal cancer statistics, 2020. CA Cancer J Clin.

[2] Zheng RS, Sun KX, Zhang SW, Zeng HM, Zou XN, Chen R (2019). [Report of cancer epidemiology in China, 2015]. Zhonghua Zhong Liu Za Zhi.

[3] Han D, Wang M, Ma N, Xu Y, Jiang Y, Gao X (2015). Long noncoding RNAs: novel players in colorectal cancer. Cancer Lett.

[4] Schmitt AM, Chang HY (2016). Long noncoding RNAs in cancer pathways. Cancer Cell.

[5] Wang Y, Lu JH, Wu QN, Jin Y, Wang DS, Chen YX (2019). LncRNA LINRIS stabilizes IGF2BP2 and promotes the aerobic glycolysis in colorectal cancer. Mol Cancer.

[6] Xu M, Xu X, Pan B, Chen X, Lin K, Zeng K (2019). LncRNA SATB2-AS1 inhibits tumor metastasis and affects the tumor immune cell microenvironment in colorectal cancer by regulating SATB2. Mol Cancer.

[7] Lukong KE, Chang KW, Khandjian EW, Richard S (2008). RNA-binding proteins in human genetic disease. Trends Genet.

[8] Huang J, Zhang A, Ho TT, Zhang Z, Zhou N, Ding X (2016). Linc-RoR promotes c-Myc expression through hnRNP I and AUF1. Nucleic Acids Res.

[9] Statello L, Guo CJ, Chen LL, Huarte M (2021). Gene regulation by long non-coding RNAs and its biological functions. Nat Rev Mol Cell Biol.

[10] Li XL, Subramanian M, Jones MF, Chaudhary R, Singh DK, Zong X (2017). Long noncoding RNA PURPL suppresses basal p53 levels and promotes tumorigenicity in colorectal cancer. Cell Rep..

[11] Hacisuleyman E, Goff LA, Trapnell C, Williams A, Henao-Mejia J, Sun L (2014). Topological organization of multichromosomal regions by the long intergenic noncoding RNA Firre. Nat Struct Mol Biol.

[12] Meng X, Fang E, Zhao X, Feng J (2020). Identification of prognostic long noncoding RNAs associated with spontaneous regression of neuroblastoma. Cancer Med.

[13] Shi X, Cui Z, Liu X, Wu S, Wu Y, Fang F (2019). LncRNA FIRRE is activated by MYC and promotes the development of diffuse large B-cell lymphoma via Wnt/beta-catenin signaling pathway. Biochem Biophys Res Commun.

[14] Takahashi H, Nishimura J, Kagawa Y, Kano Y, Takahashi Y, Wu X (2015). Significance of polypyrimidine tract-binding protein 1 expression in colorectal cancer. Mol Cancer Ther.

[15] Sun X, Haider Ali MSS, Moran M (2017). The role of interactions of long non-coding RNAs and heterogeneous nuclear ribonucleoproteins in regulating cellular functions. Biochem J.

[16] Li B, Yen TS (2002). Characterization of the nuclear export signal of polypyrimidine tract-binding protein. J Biol Chem.

[17] Ghetti A, Pinol-Roma S, Michael WM, Morandi C, Dreyfuss G, hnRNP I (1992). the polypyrimidine tract-binding protein: distinct nuclear localization and association with hnRNAs. Nucleic Acids Res.

[18] Mah LY, Ryan KM (2012). Autophagy and cancer. Cold Spring Harb Perspect Biol.

[19] Chen N, Debnath J (2010). Autophagy and tumorigenesis. FEBS Lett.

[20] Roy S, Debnath J (2010). Autophagy and tumorigenesis. Semin Immunopathol.

[21] Hu F, Li G, Huang C, Hou Z, Yang X, Luo X (2020). The autophagy-independent role of BECN1 in colorectal cancer metastasis through regulating STAT3 signaling pathway activation. Cell Death Dis.

[22] Zhang MY, Gou WF, Zhao S, Mao XY, Zheng ZH, Takano Y (2014). Beclin 1 expression is closely linked to colorectal carcinogenesis and distant metastasis of colorectal carcinoma. Int J Mol Sci.

[23] Berezovskii ME (1978). [Method of staining of semi-thin sections with hematoxylin-eosin]. Arkh Patol.

[24] Li G, Jiang H, Zheng C, Zhu G, Xu Y, Sheng X (2017). Long noncoding RNA MRAK009713 is a novel regulator of neuropathic pain in rats. Pain.

[25] Sawicka K, Bushell M, Spriggs KA, Willis AE (2008). Polypyrimidine-tract-binding protein: a multifunctional RNA-binding protein. Biochem Soc Trans.

[26] Takahashi K, Yan I, Haga H, Patel T (2014). Long noncoding RNA in liver diseases. Hepatology.

[27] Li M, Zhao LM, Li SL, Li J, Gao B, Wang FF (2018). Differentially expressed lncRNAs and mRNAs identified by NGS analysis in colorectal cancer patients. Cancer Med.

[28] Djebali S, Davis CA, Merkel A, Dobin A, Lassmann T, Mortazavi A (2012). Landscape of transcription in human cells. Nature.

[29] Derrien T, Johnson R, Bussotti G, Tanzer A, Djebali S, Tilgner H (2012). The GENCODE v7 catalog of human long noncoding RNAs: analysis of their gene structure, evolution, and expression. Genome Res.

[30] Dimitrova N, Zamudio JR, Jong RM, Soukup D, Resnick R, Sarma K (2014). LincRNA-p21 activates p21 in cis to promote Polycomb target gene expression and to enforce the G1/S checkpoint. Mol Cell.

[31] Carpenter S, Aiello D, Atianand MK, Ricci EP, Gandhi P, Hall LL (2013). A long noncoding RNA mediates both activation and repression of immune response genes. Science.

[32] Romanelli MG, Weighardt F, Biamonti G, Riva S, Morandi C (1997). Sequence determinants for hnRNP I protein nuclear localization. Exp Cell Res.

[33] Cote CA, Gautreau D, Denegre JM, Kress TL, Terry NA, Mowry KL (1999). A Xenopus protein related to hnRNP I has a role in cytoplasmic RNA localization. Mol Cell.

[34] Romanelli MG, Diani E, Lievens PM (2013). New insights into functional roles of the polypyrimidine tract-binding protein. Int J Mol Sci.

[35] Liu J, Li Y, Tong J, Gao J, Guo Q, Zhang L (2018). Long non-coding RNA-dependent mechanism to regulate heme biosynthesis and erythrocyte development. Nat Commun.

[36] Galluzzi L, Pietrocola F, Bravo-San Pedro JM, Amaravadi RK, Baehrecke EH, Cecconi F (2015). Autophagy in malignant transformation and cancer progression. EMBO J.

[37] Amaravadi RK, Kimmelman AC, Debnath J (2019). Targeting autophagy in cancer: recent advances and future directions. Cancer Disco.

[38] Jo YK, Roh SA, Lee H, Park NY, Choi ES, Oh JH (2017). Polypyrimidine tract-binding protein 1-mediated down-regulation of ATG10 facilitates metastasis of colorectal cancer cells. Cancer Lett.

[39] Han M, Qian X, Cao H, Wang F, Li X, Han N (2020). lncRNA ZNF649-AS1 induces trastuzumab resistance by promoting ATG5 expression and autophagy. Mol Ther.

