# Supplementary material for: LncRNA FIRRE functions as a tumor promoter by interaction with PTBP1 to stabilize BECN1 mRNA and facilitate autophagy
Source: Cell Death Dis. 2022 Feb 2;13(2):98. doi: 10.1038/s41419-022-04509-1 (PMC8811066; doi:10.1038/s41419-022-04509-1)
Supplement: Supplementary file 1 — Supplement [file 41419_2022_4509_MOESM1_ESM.docx]

Table 1 All primer sequences

| Gene | sequence |
| --- | --- |
| GAPDH | F:5’-ATGGTGGTGAAGACGCCAGTA-3’ |
|  | R:5-’GGCACAGTCAAGGCTGAGAATG-3’ |
| FIRRE | F:5’-CTGTGACCTCGCTTCACTTCT-3’ |
|  | R:5’-GTGGCAAAGAGCAGAAGATAGA-3’ |
| PTBP1 | F:5’ -GAAGGACCGCAAGATGGCACTG-3’ |
|  | R:5’- TAGATGGTGGACTTGGAGAAGGAGAC-3’ |
| BECN1 | F:5’ -GGAGCTGCCGTTATACTGTTCTGG-3’ |
|  | R:5’-TGCCTCCTGTGTCTTCAATCTTGC-3’ |

Table2 Sequence of siRNA for PTBP1 knockdown, ASO for FIRRE knockdown, RNA probes for FIRRE FISH

|  | | | sequence |
| --- | --- | --- | --- |
| siPTBP1 | Si1 | sense | 5’-GCACAGUGUUGAAGAUCAUTT-3’ |
|  |  | antisense | 5’-AUGAUCUUCAACACUGUGCTT-3’ |
|  | Si2 | sense | 5’-GCCUCAACGUCAAGUACAATT-3’ |
|  |  | antisense | 5’-UUGUACUUGACGUUGAGGCTT-3’ |
|  | Si3 | sense | 5’-CCCUCAUUGACCUGCACAATT-3’ |
|  |  | antisense | 5’-UUGUGCAGGUCAAUGAGGGTT-3’ |
| Target sequence of ASO in FIRRE | | | CTGAAAATGAAGGCCAATAC |
| RNA probes for FIRRE FISH | | | ATGGTGTTTGCAAGCCAGGTACAGT |
|  |  |  | GCAGTGACTCTGCTACCACAAAGGT |
|  |  |  | ATATGTCGTGAAATATGCTTCAGAA |

Table 3 Characteristic of patients from TCGA data

| Variable | | Numbers | ratio（%） |
| --- | --- | --- | --- |
| Sex | Male | 329 | 75.6 |
|  | Female | 156 | 24.4 |
| Age | ≤65 | 196 | 45.1 |
|  | ＞65 | 239 | 54.9 |
| Stage | I | 55 | 12.6 |
|  | II | 129 | 29.7 |
|  | III | 181 | 41.6 |
|  | IV | 43 | 9.9 |
|  | Unknown | 27 | 6.2 |
| T stage | T1 | 21 | 4.8 |
|  | T2 | 89 | 20.4 |
|  | T3 | 196 | 45.1 |
|  | T4 | 119 | 27.4 |
|  | Unknown | 10 | 2.3 |
| N stage | N0 | 128 | 29.4 |
|  | N1 | 117 | 27.0 |
|  | N2 | 84 | 19.3 |
|  | N3 | 87 | 20.0 |
|  | Unknown | 19 | 43.7 |
| M stage | M0 | 385 | 88.5 |
|  | M1 | 30 | 6.9 |
|  | Unknown | 20 | 4.6 |

**Legends**

**Fig S1 FIRRE has no association with age and gender.** **a.** FIRRE level is not related to the age. **b.** FIRRE level is not associated with the sex.

**Fig S2 Efficiency of siPTBP1 in cells. a.** In RKO cell, the efficiency of siRNAs targeted PTBP1 was detected by qRT-PCR, indicating the decrease of PTBP1 at RNA level. **b.** The siPTBP1s significantly decrease the level of PTBP1 compared with the control siRNA in HCT116 cells. **c.** According to the high efficiency of siPTBP1s, si3-mediated knock-down of PTBP1 was detected by WB. The result shows that si3 reduces the expression of PTBP1. Bars, ±SD; Statistical analysis: ANOVA test, t tests. * p<0.01; **p<0.001; ****p*<0.0001

**Fig S3** **The cells were treated with actinomycin D**. RKO(**a**) and HCT116(**b**) cells transfected with siPTBP1 or ASO-FIRRE alone or in combination were treated with actinomycin D (5 μg/ml). RNA was extracted at 60 mins, and qPCR was used to quantify BECN1 mRNA. All experiments were repeated double times or more. Statistical analysis: ANOVA test. * p<0.01; **p<0.001; ****p*<0.0001
