# Supplementary figures and images for: LncRNA FIRRE functions as a tumor promoter by interaction with PTBP1 to stabilize BECN1 mRNA and facilitate autophagy
Source: Cell Death Dis. 2022 Feb 2;13(2):98. doi: 10.1038/s41419-022-04509-1 (PMC8811066; doi:10.1038/s41419-022-04509-1)

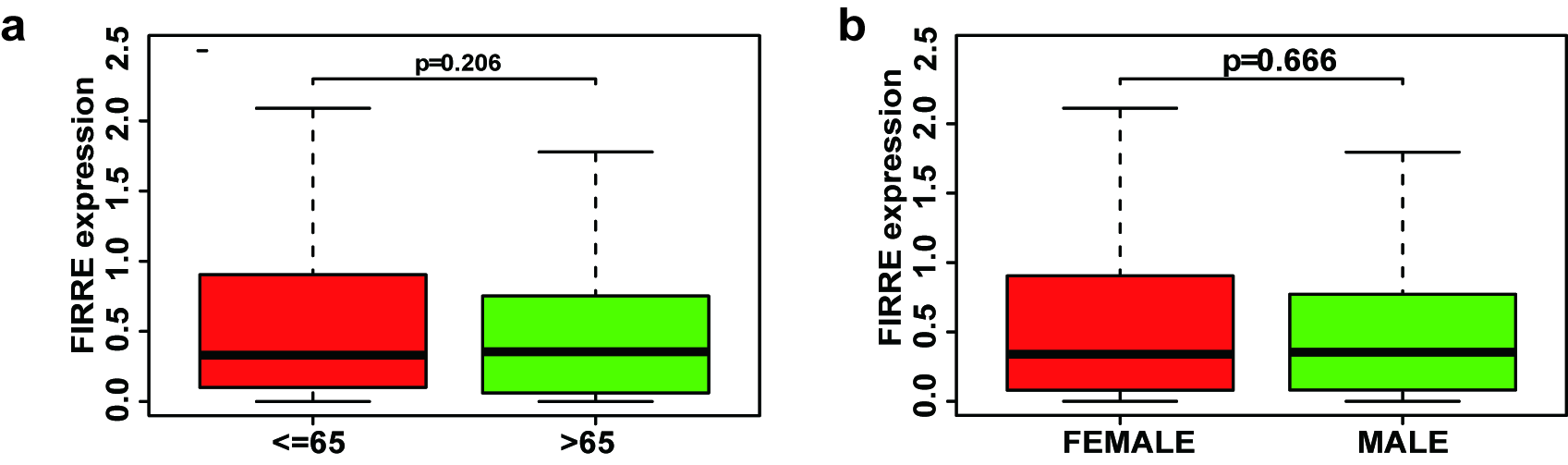

Supplement: Supplementary file 2 — FigS1 [file 41419_2022_4509_MOESM2_ESM.tif]

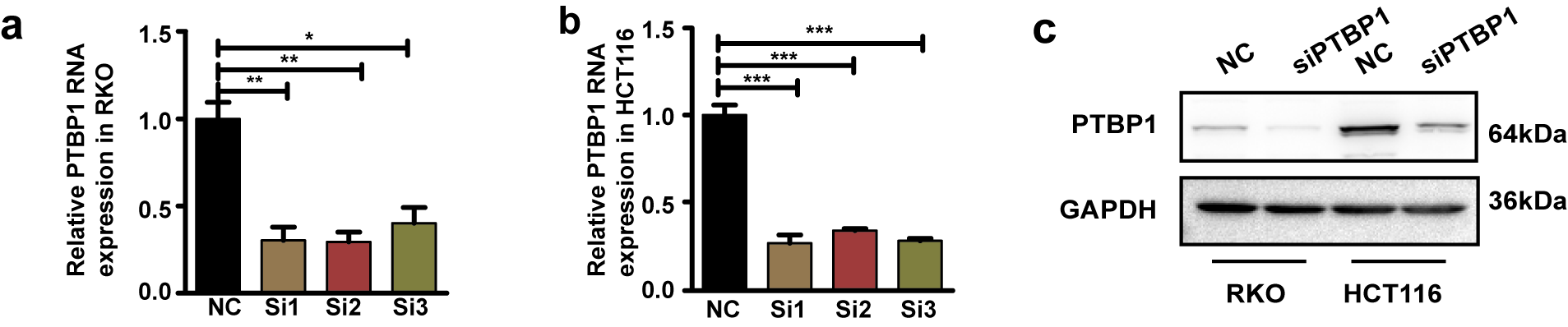

Supplement: Supplementary file 3 — FIG S2 [file 41419_2022_4509_MOESM3_ESM.tif]

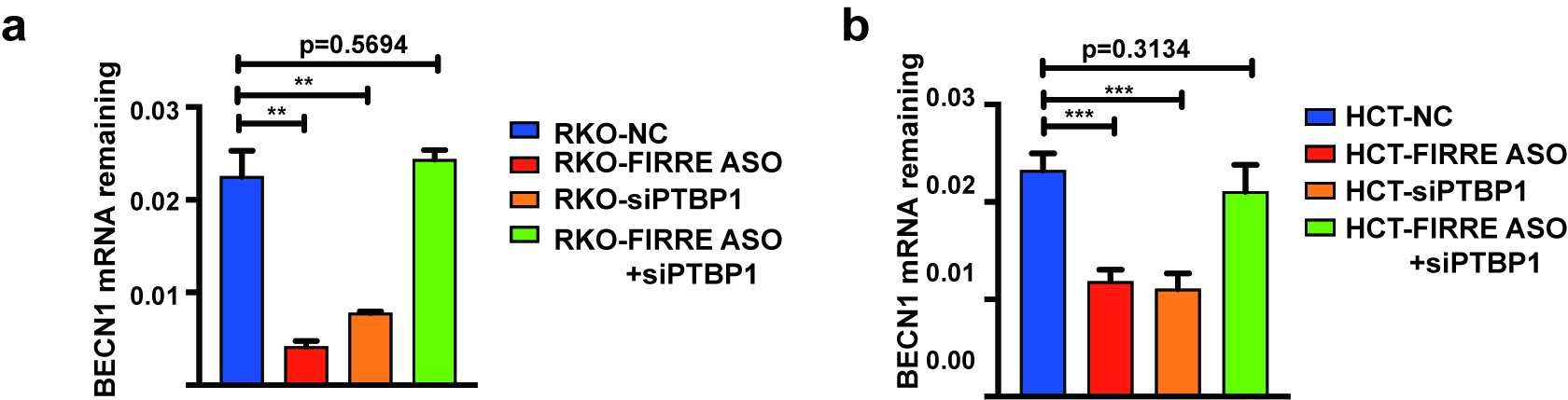

Supplement: Supplementary file 4 — Fig S3 [file 41419_2022_4509_MOESM4_ESM.tif]
