# Supplementary material for: LncRNA FIRRE functions as a tumor promoter by interaction with PTBP1 to stabilize BECN1 mRNA and facilitate autophagy
Source: Cell Death Dis. 2022 Feb 2;13(2):98. doi: 10.1038/s41419-022-04509-1 (PMC8811066; doi:10.1038/s41419-022-04509-1)
Supplement: Supplementary file 5 — Related Manuscript File [file 41419_2022_4509_MOESM5_ESM.docx]

**Authors’ contribution statement**

YW, MJ and XF conceived and designed the experiments; YW, SZ, and ZL performed the experiments; WL and YW analyzed the data; WL, MC and SZ contributed with reagents and patient material; and YW and XF wrote the paper. All authors read and approved the final manuscript.
