# Supplementary material for: LncRNA FIRRE functions as a tumor promoter by interaction with PTBP1 to stabilize BECN1 mRNA and facilitate autophagy
Source: Cell Death Dis. 2022 Feb 2;13(2):98. doi: 10.1038/s41419-022-04509-1 (PMC8811066; doi:10.1038/s41419-022-04509-1)
Supplement: Supplementary file 6 — Related Manuscript File [file 41419_2022_4509_MOESM6_ESM.docx]

Title and running title

Title. Long non-coding RNA FIRRE-dependent mechanism to regulate the progression of CRC

Running title Function and mechanism of FIRRE in colorectal cancer
