# Supplementary material for: LncRNA FIRRE functions as a tumor promoter by interaction with PTBP1 to stabilize BECN1 mRNA and facilitate autophagy
Source: Cell Death Dis. 2022 Feb 2;13(2):98. doi: 10.1038/s41419-022-04509-1 (PMC8811066; doi:10.1038/s41419-022-04509-1)
Supplement: Supplementary file 8 — Related Manuscript File [file 41419_2022_4509_MOESM8_ESM.docx]

Email address

Yajie Wang [YajieWang911119@126.com](mailto:YajieWang911119@126.com)

Miao Jiang  jiangmiao1978@163.com

Xiaoming Fan [xiaomingfan57@hotmail.com](mailto:xiaomingfan57@hotmail.com)
