# Supplementary material for: LncRNA FIRRE functions as a tumor promoter by interaction with PTBP1 to stabilize BECN1 mRNA and facilitate autophagy
Source: Cell Death Dis. 2022 Feb 2;13(2):98. doi: 10.1038/s41419-022-04509-1 (PMC8811066; doi:10.1038/s41419-022-04509-1)
Supplement: Supplementary file 9 — Related Manuscript File [file 41419_2022_4509_MOESM9_ESM.docx]

Work telephone numbers

Yajie Wang +86-187-2101-8493

Miao Jiang +86-189-3081-7112

Xiaoming Fan +86-189-3081-9188
