# Supplementary material for: LncRNA FIRRE functions as a tumor promoter by interaction with PTBP1 to stabilize BECN1 mRNA and facilitate autophagy
Source: Cell Death Dis. 2022 Feb 2;13(2):98. doi: 10.1038/s41419-022-04509-1 (PMC8811066; doi:10.1038/s41419-022-04509-1)
Supplement: Supplementary file 10 — Related Manuscript File [file 41419_2022_4509_MOESM10_ESM.docx]

Institutions

Yajie Wang Department of Gastroenterology, Jinshan Hospital, Fudan University, 1508 Longhang Road, Shanghai, 201508, China.

Zhengyang LI Department of Gastroenterology, Jinshan Hospital, Fudan University, 1508 Longhang Road, Shanghai, 201508, China.

Shizan Xu. Department of Gastroenterology, Jinshan Hospital, Fudan University, 1508 Longhang Road, Shanghai, 201508, China.

Wenjun Li Department of Anaesthesiology, Shanghai Public Health Clinical Center, Fudan University, 2901 Caolang Road, Shanghai, 201503, China.

Mengyun Chen General Practice of Huamu Community Health Service Center, 90 Yulan Road, Shanghai, 201204, China.

Miao Jiang Department of Gastroenterology, Jinshan Hospital, Fudan University, 1508 Longhang Road, Shanghai, 201508, China.

Xiaoming Fan Department of Gastroenterology, Jinshan Hospital, Fudan University, 1508 Longhang Road, Shanghai, 201508, China.
