# Supplementary material for: LncRNA FIRRE functions as a tumor promoter by interaction with PTBP1 to stabilize BECN1 mRNA and facilitate autophagy
Source: Cell Death Dis. 2022 Feb 2;13(2):98. doi: 10.1038/s41419-022-04509-1 (PMC8811066; doi:10.1038/s41419-022-04509-1)
Supplement: Supplementary file 11 — Related Manuscript File [file 41419_2022_4509_MOESM11_ESM.docx]

Full postal address

Yajie Wang Department of Gastroenterology, Jinshan Hospital, Fudan University, 1508 Longhang Road, Shanghai, 201508, China.

Miao Jiang Department of Gastroenterology, Jinshan Hospital, Fudan University, 1508 Longhang Road, Shanghai, 201508, China.

Xiaoming Fan Department of Gastroenterology, Jinshan Hospital, Fudan University, 1508 Longhang Road, Shanghai, 201508, China.
