# Supplementary material for: LncRNA FIRRE functions as a tumor promoter by interaction with PTBP1 to stabilize BECN1 mRNA and facilitate autophagy
Source: Cell Death Dis. 2022 Feb 2;13(2):98. doi: 10.1038/s41419-022-04509-1 (PMC8811066; doi:10.1038/s41419-022-04509-1)
Supplement: Supplementary file 12 — Related Manuscript File [file 41419_2022_4509_MOESM12_ESM.docx]

First and last names

First name: Yajie Last name: Wang

First name: Miao Last name: Jiang

First name: Zhengyang Last name: Li

First name: Shizan Last name: Xu

First name: Wenjun Last name: Li

First name: Mengyun Last name: Chen

First name: Xiaoming Last name: Fan
